# *Ab Initio* Modeling and Experimental Assessment of Janus Kinase 2 (JAK2) Kinase-Pseudokinase Complex Structure

**DOI:** 10.1371/journal.pcbi.1003022

**Published:** 2013-04-04

**Authors:** Xiaobo Wan, Yue Ma, Christopher L. McClendon, Lily Jun-shen Huang, Niu Huang

**Affiliations:** 1 Graduate School in Peking Union Medical College and Chinese Academy of Medical Sciences, Beijing, China; 2 National Institute of Biological Sciences, Beijing, Zhongguancun Life Science Park, Changping District, Beijing, China; 3 Department of Cell Biology, University of Texas Southwestern Medical Center at Dallas, Dallas, Texas, United States of America; 4 Skaggs School of Pharmacy and Pharmaceutical Sciences, University of California San Diego, San Diego, California, United States of America; Florida State University, United States of America

## Abstract

The Janus Kinase 2 (JAK2) plays essential roles in transmitting signals from multiple cytokine receptors, and constitutive activation of JAK2 results in hematopoietic disorders and oncogenesis. JAK2 kinase activity is negatively regulated by its pseudokinase domain (JH2), where the gain-of-function mutation V617F that causes myeloproliferative neoplasms resides. In the absence of a crystal structure of full-length JAK2, how JH2 inhibits the kinase domain (JH1), and how V617F hyperactivates JAK2 remain elusive. We modeled the JAK2 JH1–JH2 complex structure using a novel informatics-guided protein-protein docking strategy. A detailed JAK2 JH2-mediated auto-inhibition mechanism is proposed, where JH2 traps the activation loop of JH1 in an inactive conformation and blocks the movement of kinase αC helix through critical hydrophobic contacts and extensive electrostatic interactions. These stabilizing interactions are less favorable in JAK2-V617F. Notably, several predicted binding interfacial residues in JH2 were confirmed to hyperactivate JAK2 kinase activity in site-directed mutagenesis and BaF3/EpoR cell transformation studies. Although there may exist other JH2-mediated mechanisms to control JH1, our JH1–JH2 structural model represents a verifiable working hypothesis for further experimental studies to elucidate the role of JH2 in regulating JAK2 in both normal and pathological settings.

## Author Summary

Protein-protein interactions (PPIs) are essential to cellular signal transduction, and structural information about PPIs is crucial for understanding of how cellular machinery functions at the atomistic level. However, both experimental structural determination and computational prediction of PPI are challenging. In the cytoplasmic tyrosine kinase JAK2, a pseudokinase domain (JH2) negatively regulates kinase activity of its adjacent catalytic kinase domain (JH1). A gain-of-function mutation within JH2 is found in the majority of patients with myeloproliferative neoplasms, and is sufficient to cause similar diseases in murine models. Here we combined an informatics-guided protein-protein docking method with molecular dynamics simulation to construct and refine the JAK2 JH1–JH2 complex, and validated our model with mutational studies. Our modeled structure suggests that JH2 auto-inhibits JAK2 kinase activities by blocking the movements of the activation loop and the αC helix of JH1, but awaits further validation by a detailed structure of the full-length JAK2 protein.

## Introduction

Janus tyrosine kinase 2 (JAK2) belongs to the JAK family of intracellular non-receptor tyrosine kinases, which mediates signaling from a plethora of cytokine receptors [1]. Like other JAK members, JAK2 is kept inactive in the basal state. Dimerization/oligomerization of cytokine receptors upon cytokine engagement triggers trans-phosphorylation of JAK2 proteins bound to the receptor cytosolic domain, activating JAK2 kinase activity. Activated JAK2 in turn phosphorylates the cytokine receptor cytoplasmic domains to create sites of interaction for downstream signaling molecules such as the STAT (signal transduction and transcription) family of transcription factors. Constitutive activation of JAK2 either by chromosomal translocation or by gain-of-function mutations results in hematological malignancies including leukemias and myeloproliferative neoplasms (MPN) [2]–[5]. Constitutive activation of the JAK2/STAT3 pathway was also shown to be essential for the growth of human solid tumor xenografts [6]. As a consequence, JAK2 has emerged as a promising target for anti-cancer therapy. However, mechanisms underlying how JAK2 kinase activity is kept off in the basal state and turned on under normal or pathological conditions are not fully understood.

**Figure 1:**
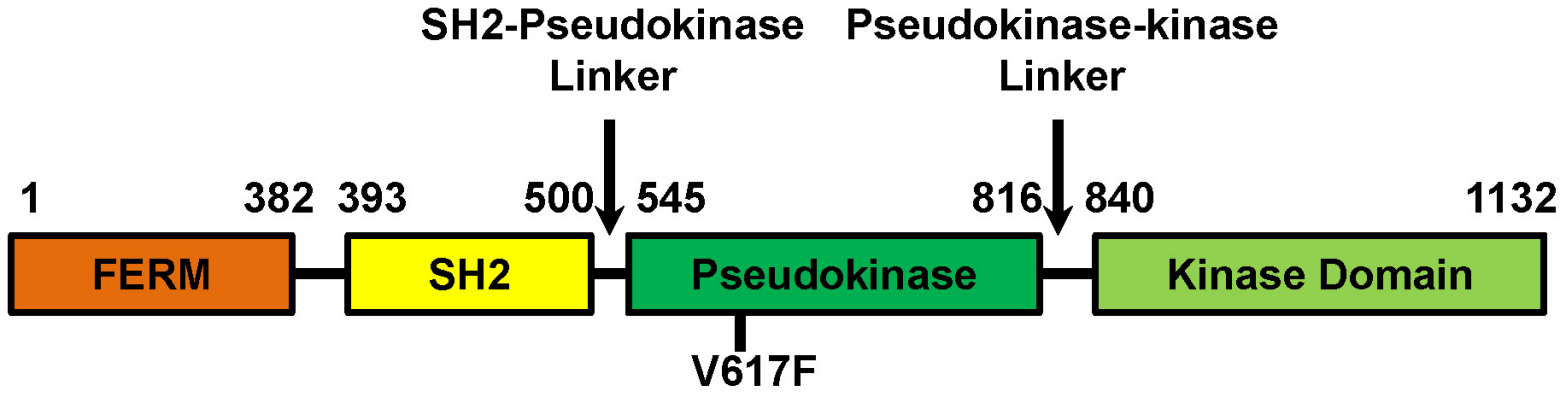
The domain organization of full length JAK2.

Strong evidence suggests that the C-terminal kinase domain (JH1, standing for JAK homology domain 1) of JAK2 is allosterically regulated by other JAK2 domains, namely a N-terminal FERM (band 4.1, ezrin, radixin, moesin) domain which associates with cytokine receptors, a Src homology-2 (SH2) domain whose function remains unclear, and a pseudokinase domain (JH2, standing for JAK homology domain 2) (Figure 1). JH2, originally thought of as a “pseudo kinase” that has a kinase fold but is devoid of kinase activity, plays particularly important roles in regulating JAK2 kinase activity. First, JH2 is essential to inhibit JH1 in the basal state. JH2 can bind to and inhibit JH1 *in trans*, and deletion of JH2 increases basal JAK2 kinase activity [7]–[9]. Recent studies also demonstrated that JH2 actually possesses low catalytic activity and autophosphorylates two negative regulatory sites in the SH2-JH2 domain linker and in JH2 to maintain basal auto-inhibition [10]. Functional importance of JH2-mediated auto-inhibition is underscored by the existence of a hyperactivating JAK2 mutation therein in MPN patients. This hyperactivating mutation, V617F, is found in almost all MPN patients with polycythemia vera and is sufficient to cause a similar disease in mice [11], [12]. The V617F mutation is also frequently found in essential thrombocythemia and primary myelofibrosis, two other kinds of MPN. Second, we and others have shown that JH2 also positively regulates JAK2 kinase activity as it is essential for cytokine-induced JAK2 activation. Deletion or mutations in JH2 resulted in JAK2 variants that showed elevated basal activity but cannot be further stimulated by cytokine, and that the elevated activity is below that of cytokine-stimulated wild-type JAK2 (JAK2-WT) [8], [12]. In addition, we showed that the JAK2-V617F mutant has a lower K_m_ for substrates compared to JAK2-WT, indicating that JH2 can also promote substrate binding to JH1 [13].

Understanding the exact molecular mechanisms underlying how JH2 both positively and negatively regulates JH1 calls for a structure of the full-length JAK2. Unfortunately, despite many years of efforts in the field, such a structure is not yet available. Computational modeling thus represents an important technique in bridging the gap to explore the relationship between structure and function. The two hypotheses that JH2 negatively regulates JAK2 kinase activity, either by binding and inhibiting JH1 directly or via the two negative regulatory phosphorylation sites, are not mutually exclusive. In addition, the latter hypothesis may be more complex to involve other JAK2 domains. We thus chose to start our interrogation of JH2-mediated regulation of JAK2 kinase activity from modeling the JH1–JH2 complex. As both JH1 and JH2 adopt a kinase fold, one practical strategy is to model the JH1–JH2 complex using dimeric kinase forms indicated by crystal structures of kinase complexes or by assembly derived from crystal packing [14]. For EGFR [15] and PKR [16], [17], the dimeric unit implicated by crystal packing helped to reveal novel dimerization and activation mechanisms. A structure model of JAK2, based on the dimeric form of FGFR1 kinase implicated by crystal packing [18], was built by Kroemer and coworkers [19], [20]. In this model, two interfaces between JH1 and JH2 were proposed: one dominated by interactions between the two paralleled αC helices, and the other between the JH1 activation loop and a loop in JH2 that includes V617. The V617F mutation was proposed to destabilize the latter interface to relieve auto-inhibition [21]. Subsequently, Lee and coworkers performed molecular dynamics (MD) simulations on this model to explore the hypothesized conformational changes at the atomic level and found a strong π-π stacking interaction between V617F and F595 [22]. The importance of this interaction in the ability of V617F to constitutively activate JAK2 was later validated experimentally [23], [24].

**Figure 2:**
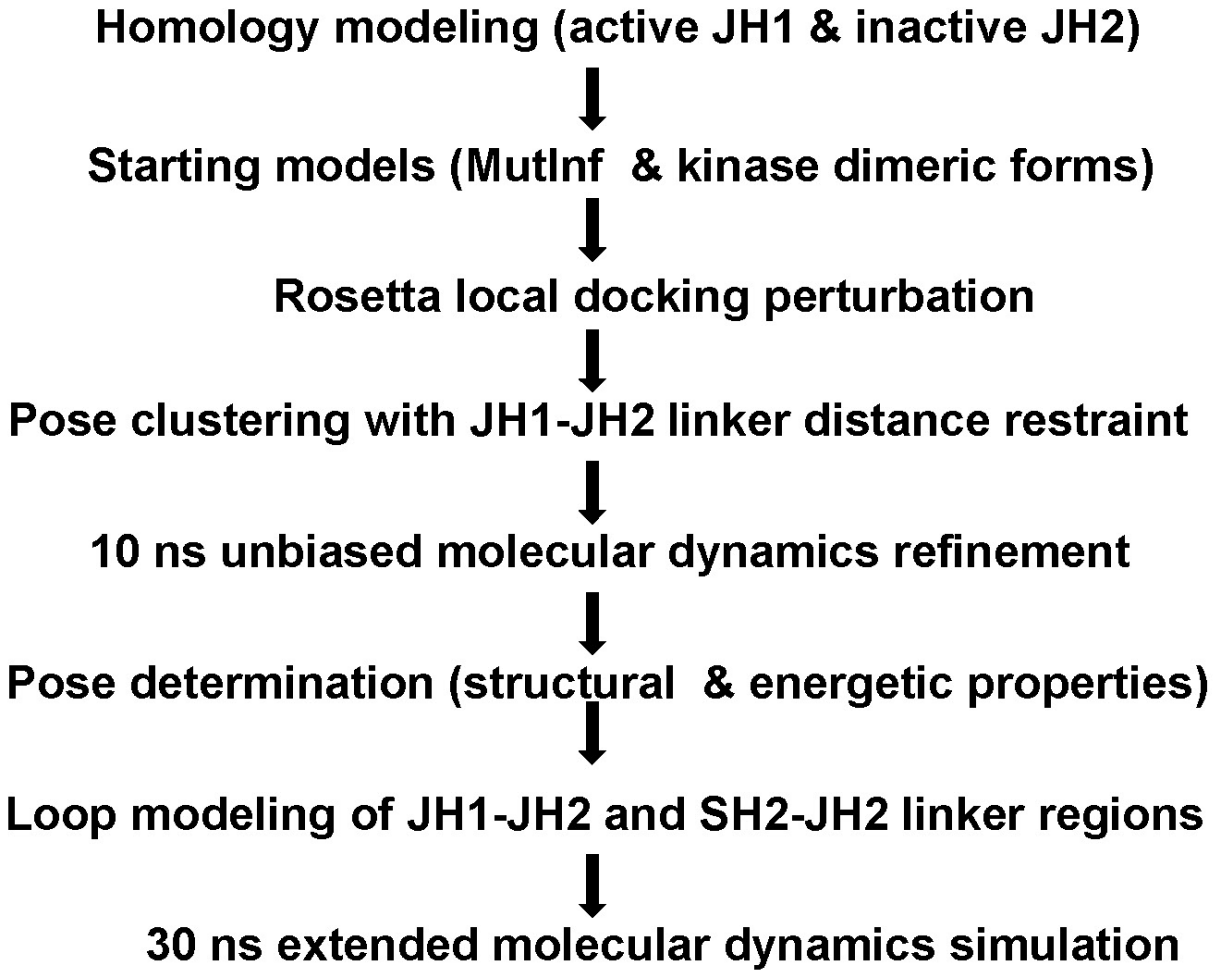
The schematic of hierarchical protein-protein docking procedure.

Protein kinases adopt at least two distinct conformational states: a structurally-conserved “on” state that is active and a less-structurally-conserved “off” state that has minimal activity [25]. The plethora of kinase crystal structures to date have provided a structural basis for kinase regulatory mechanisms, where conformational changes in the αC helix and in the activation loop are the common features [25]. For example, the fibroblast growth factor receptor 2 (FGFR2) has an autoinhibitory “molecular brake” involving movements of the activation loop, the αC helix and the kinase hinge region [26]. The autoinhibitory domain of AMP-activated protein kinase (AMPK) constrains the mobility of αC helix and results in much lower kinase activity [27]. In Kroemer's model, both JH1 and JH2 domains were built in an inactive conformation. In addition, this model was minimally refined, and conformational stability was not evaluated. In light of recent findings that the JAK2 JH2 domain actually possesses kinase activity and phosphorylates two negative regulatory sites, a revised model is warranted. We thus developed a novel step-wise computational strategy to build a new model *ab initio*, with JH2 in an active conformation and JH1 in an inactive conformation. In this paper we describe a hierarchical protein-protein docking and refinement protocol (Figure 2) and report the most energetically favorable and structurally stable model of the JAK2 JH1–JH2 complex. In our model, JH2 stabilizes the inactive conformation of JH1 through extensive hydrophobic contacts and electrostatic interactions. Importantly, we experimentally assessed and validated critical interfacial residues predicted from our model, which would not have been envisioned from previous models. Although we cannot rule out the possibility that dimerization and autophosphorylation of JH2 might be the predominant mechanism to inhibit JH1, our JH1–JH2 model represents a verifiable working hypothesis for further experimental studies, with the ultimate goal to understand how JH2 regulates JAK2 in both normal and pathological settings.

## Results

### Identify potential allosteric sites using MutInf

**Figure 3:**
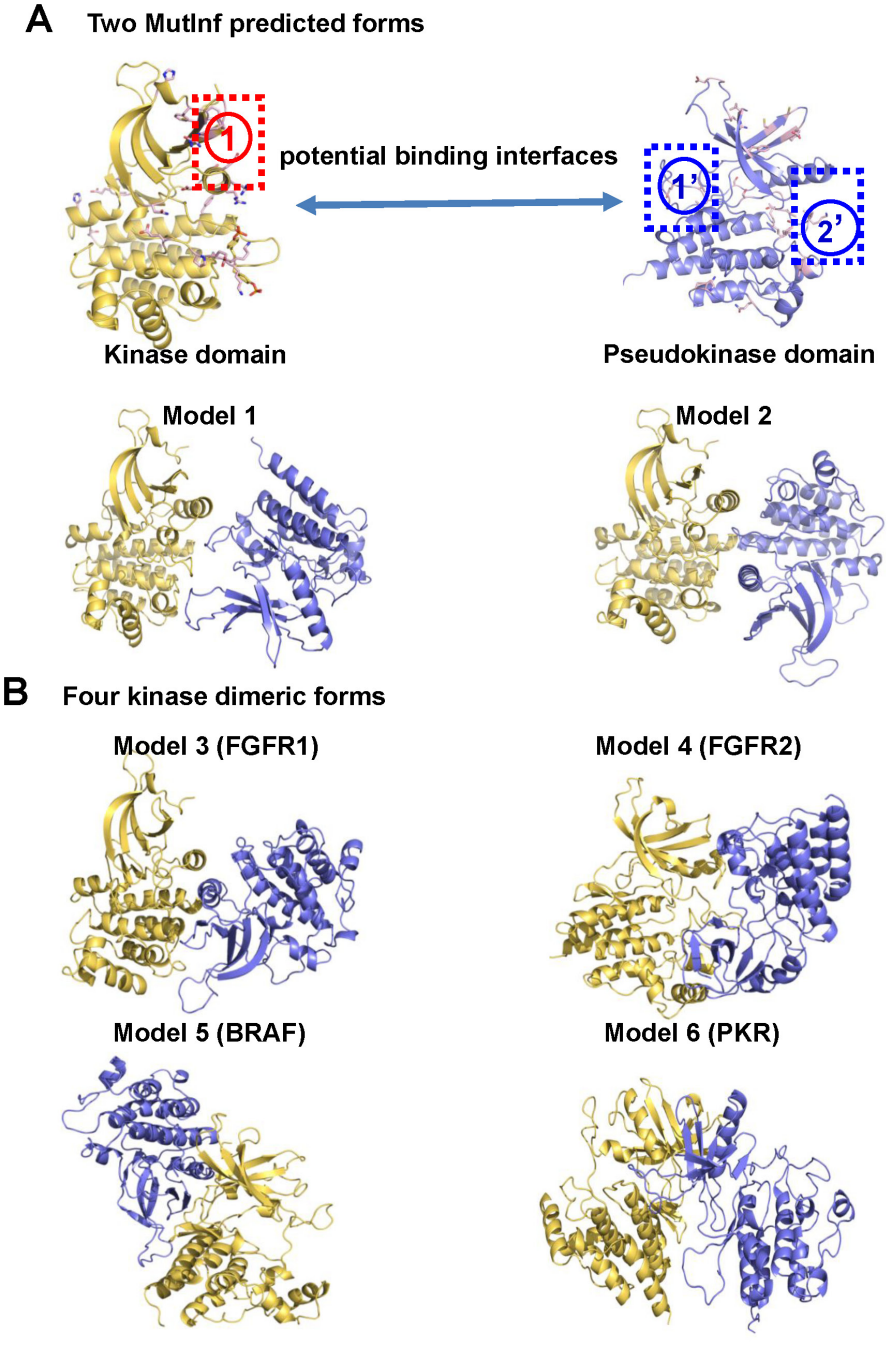
Starting models were generated by connecting sites identified by MutInf or by using dimer geometries from crystal structures. (A) Two predefined packing models were constructed by manually joining the αC helix in the JAK2 kinase domain with two highly coupled sites in the JH2 pseudokinase domain identified by MutInf. (B) Four additional packing models were built by alignment to crystal structures of kinase dimeric forms (FGFR1, FGFR2, BRAF and PKB).

Two strategies were employed to build an initial model of the complex between the inactive conformation of JH1 and the active conformation of JH2 (Figure 3). In the first strategy, models were constructed based on the hypothesis that the JH1–JH2 complex interface connects allosteric sites of JH1 and JH2. Allosteric sites in JH1 and JH2 were identified by MutInf (mutual information-based analysis of MD simulations), a novel method previously developed to identify allosteric sites in an unbiased, statistically robust manner [28]. In the second strategy, models were constructed based on available kinase dimeric forms similar to those in the study by Kroemer and coworkers [19], [20]. As detailed in the Methods section, a total of four diverse JAK2 JH1–JH2 complex structures were modeled according to the dimer structures of FGFR1 [18], FGFR2 [26], BRAF [29] and RNA-dependent protein kinase PKR [16].

**Figure 4:**
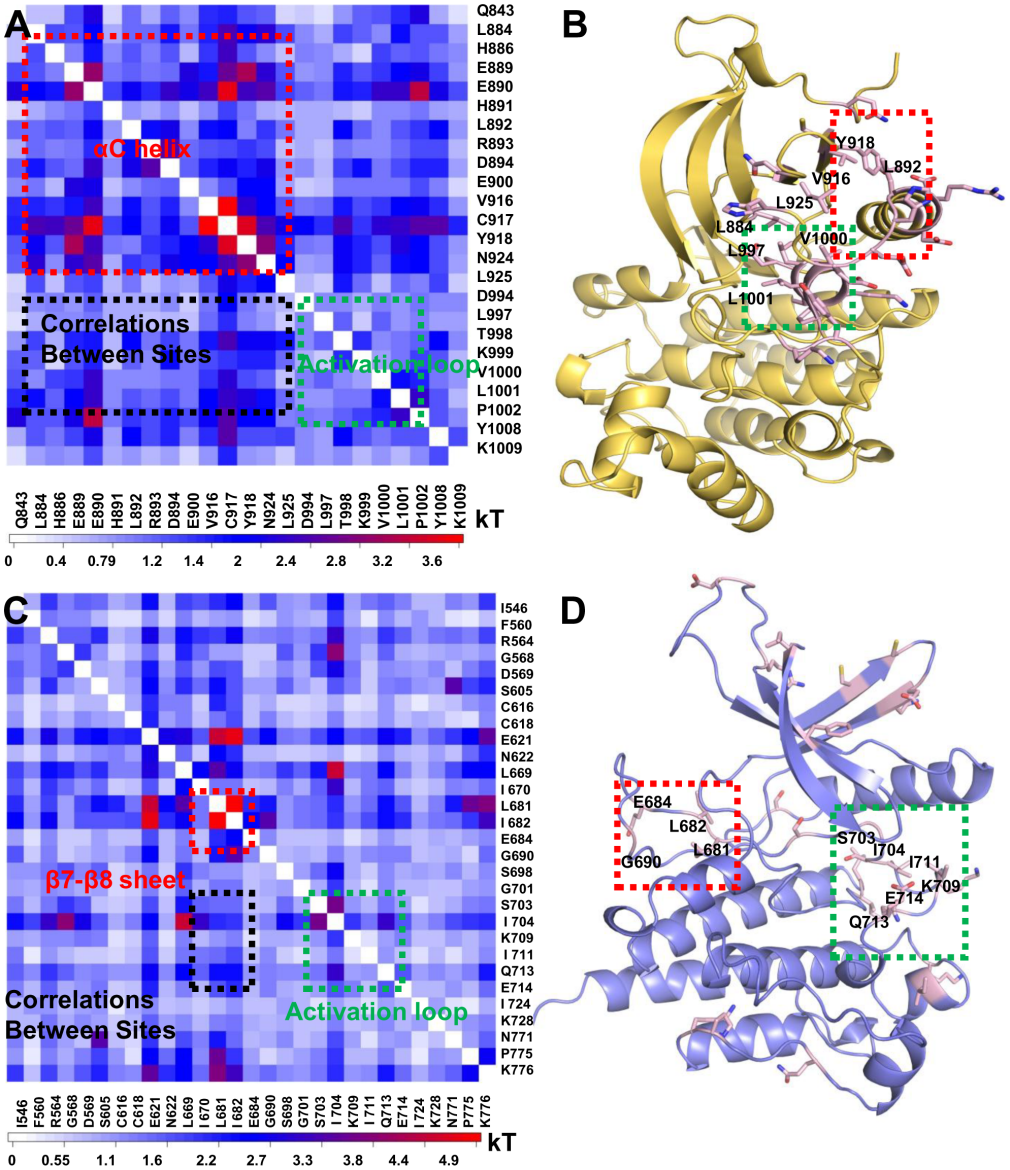
Correlated motions couple active to putative allosteric sites in the JAK2 JH1 and JH2 domains. (A) The pairwise matrix of the highly coupled residues in the inactive conformation of the JAK2 JH1 domain. (B) Strong correlations between the activation loop (green box) and the αC helix (red box) are observed in the JH1 domain (yellow in cartoon). (C) The pairwise matrix of the highly coupled residues in the active conformation of the JH2 domain. (D) The strong correlations of the loop between β7–β8 sheets near the hinge region (green box) with activation loop (red box) shown in the JH2 domain (blue in cartoon).

We first used MutInf (details in the Methods) to identify allosteric sites in the crystal structure of JH1 in the active conformation (PDB ID: 2B7A) to validate our method. Consistent with conventional kinase regulatory mechanisms, dynamical couplings from the activation loop and the hinge region to the αC helix were observed. Direct coupling between the αC helix and activation loop occurs via a polar network; for example, residues R897 in the αC helix and K1011 in the activation loop coordinating the phosphorylated Y1008. The strongest correlated residues in the pairwise matrix were mapped on to the structure (Figure S1). We next modeled JH1 in the inactive conformation based on the inactive conformation of EGFR (PDB ID: 2GS7) and examined the pattern of correlated motions using MutInf (Figures 4A–B, and S2A–B). Notably, a more robust communication between the αC helix and the activation loop was observed in the inactive form than in the active form. Unlike the polar network found in active JH1, a hydrophobic network in the inactive JH1 connects the greasy surfaces formed by the αC helix and the activation loop. In particular, a cluster of hydrophobic residues, namely L997, V1000 and L1001 in the activation loop, are coupled to the following residues: L884 in β3 sheet, L925 in β4 sheet, V916 and Y918 in the β5 sheet, and L892 in the αC helix. Other correlated residues, such as E890, H891, R893, D894 and E900 are also located in the aC helix. Therefore, conformational changes of the αC helix are highly coupled to the activation loop in JH1 in both the active and inactive states, despite the nature of the network differs.

We also modeled JH2 in the active conformation and applied MutInf analysis (Figures 4C–D, and S2C–D). Surprisingly, no significant couplings were identified for residues in the αC helix, in contrast to results from JH1. Instead, we identified two novel coupling sites: one consists of residues L681, L682, E684 and G690 in the β7–β8 sheet near the hinge region and the other is close to the catalytic loop (residues L669 and I670) and activation loop (S703, I704, K709, I711, Q713 and E714). It is likely that these two correlated sites in JH2 are involved in stabilizing JH1 in an inactive conformation.

### Refinement of JH1–JH2 complex models

Six initial models (Figure 4), four from modeling kinase dimers and two from MutInf predicted interfaces were refined by a hierarchical protein-protein docking and refinement procedure to systematically improve the quality of these complex models. Among the six models, Model 2 (derived from the MutInf approach) was the most structurally stable and energetically favorable structure throughout the entire 10 ns MD simulation (Table 1), as assessed by averaged root-mean-square fluctuation of JH2 (PK_RMSF 1.8 Å), averaged interaction energy (−217.1±26.5 kcal/mol), averaged charged and hydrophobic contacts (7 and 16 respectively), and averaged BSA (∼1300 Å^2^). Internal motions of JH2 and JH1 themselves were relatively small, with RMSF values (Cα) around 1∼2 Å among all models (data not shown). Therefore, Model 2 likely represents the near-native conformation of the JAK2 JH1–JH2 complex in an auto-inhibited state. In addition, fluctuations of the αC helix were measured to assess whether the predicted protein-protein interface in model 2 would block the mobility of αC helix (Table 1). Indeed, the motion of αC helix in Model 2 is the lowest of all models due to strong interfacial interactions between JH2 and JH1.

**Table 1:** The structural and energetic properties of six predetermined models through 10 ns MD simulation.

Type	Interaction Energy (kcal/mol)	PK_RMSF (Å)	BSA(Å^2^)	Contacts	KH_RMSF (Å)
Model (Kroemer's)	−63.0±30.6	3.0±1.0	964	2/4	1.4±0.3
Model 1 (MutInf)	−198.7±27.8	2.8±0.8	912	8/6	1.1±0.3
Model 2 (MutInf)	−217.1±26.5	1.8±0.3	1325	7/16	0.8±0.2
Model 3 (FGFR1)	−78.2±33.7	2.7±1.0	1340	0/6	1.0±0.2
Model 4 (FGFR2)	−57.5±36.1	3.2±1.0	771	3/8	1.1±0.2
Model 5 (BRAF)	−133.5±45.5	2.8±1.3	1100	6/10	1.3±0.3
Model 6 (PKR)	−121.7±54.1	2.8±0.8	889	6/6	1.3±0.3

The interaction energy is accounting the interaction energy between the kinase and pseudokinase domain. PK_RMSF is the RMSF of Cα atom of JH2 after superimposing on kinase domain in MD simulation. BSA is the buried surface areas and H/I are contacts between the hydrophobic residues within 5 Å and charged residues within 6 Å in the interfaces. KH_RMSF is the RMSF of Cα atom in kinase αC helix (residues 885 to 907) after superimposing on kinase domain in MD simulation.

### Investigation of how the V617F mutation constitutively activates JAK2

**Figure 5:**
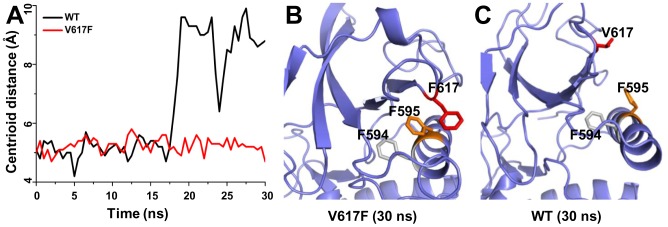
The V617F mutant in the JH2 domain packs closer to F595 than in the wild-type JH2 in 30 ns MD simulations. (A) Centroid distances between F595 and V/F617 in JAK2-WT and JAK2-V617F show a closer packing in the V617F mutant than in the wild type. (B, C) Cartoon representations are shown for the last snapshot of JAK2-V617F and wild-type JH2, respectively, after 30 ns MD simulations, showing a contact in the V617F mutant that is not present after 30 ns for the wild-type pseudokinase. The F595, V/F617 and F594 are colored in orange, red and grey.

We explored the auto-inhibition mechanism of JAK2-WT and the constitutive activation mechanism of the JAK2-V617F mutant based on model 2. We carried out an additional 30 ns unbiased MD simulations for both JAK2-WT and JAK2-V617F structures after the addition of two linkers between JH2 and JH1, and between the SH2 domain and JH2, respectively. A strong π-π stacking interaction with the neighboring residue F595 was proposed to be important for the V617F mutation to constitutively activate JAK2 [22]–[24]. Consistent with this notion, the centroid distance between residues 595 and 617 was stable across the entire 30 ns simulation in the JAK2-V617F model (Figures 5A and 5B), with the average centroid distance of 5.8 Å, which is within the range generally accepted for a π-π stacking interaction (4.5∼7.0 Å) [30]. In contrast, in the JAK2-WT model, the distance between F595 and V617 increased, accompanied by a large conformational change of the β4/β5 region after 18 ns (Figures 5A and 5C). Interestingly, we observed that a model lacking the SH2-JH2 linker also lost this π-π stacking interaction after 22 ns simulation (Figure S3), indicating that the SH2-JH2 linker may regulate JAK2 kinase activity. These results are in line with the fact that activating mutations in this linker (exon 12 mutations) are found in MPN patients [12], [31].

**Figure 6:**
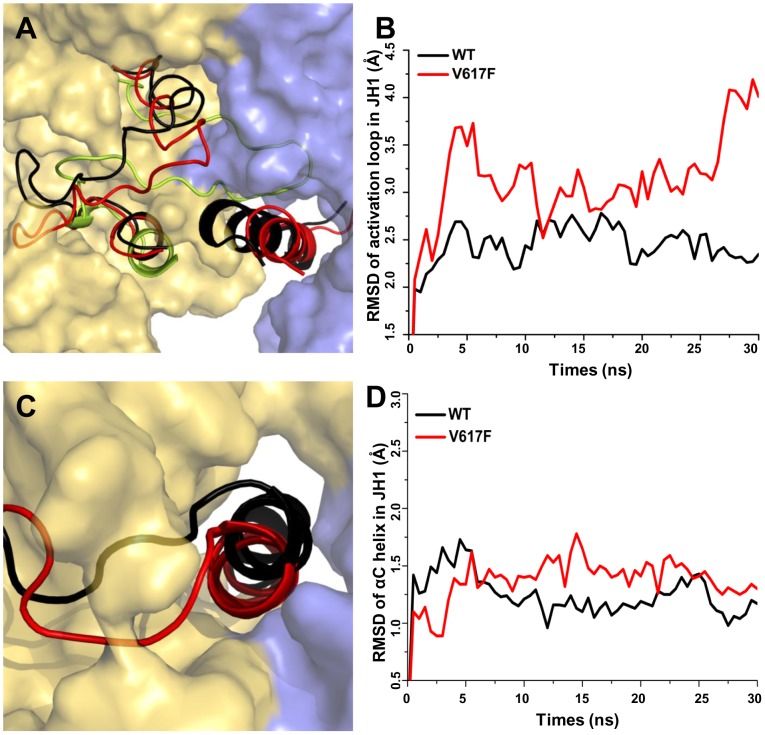
Motions of the activation loop and αC-helix in JAK2-WT and JAK2-V617F in 30 ns MD simulation. (A) The JAK2-V617F mutant increases the flexibility of the activation loop in the kinase domain by restricting the movement of αC helix region in the JH2. The activation loop in the kinase domain of JAK2-WT (black) and JAK2-V617F (red) is shown alongside the crystal structure (green) in cartoon representation using PyMOL. Other residues are shown in surface representation. (B) RMSDs of Cα atoms during 30 ns of MD simulation show displacement of the kinase domain activation loop residues 994–1028 in the V617F mutant (black) but not the wildtpype (red). (C) Conformations of the αC helix in kinase domain after 30 ns MD for V617F mutant (black) and wild type (red). (D) RMSDs for the kinase αC helix (residues 586–606) in JAK2-WT and JAK2-V617F during 30 ns MD simulation show that the position of this helix is relatively stable in both cases. RMSDs are computed over Cα atoms of JAK2 with respect to the initial model after superposition of the kinase domain.

We also examined the structural and energetic consequences introduced by the V617F mutation. In contrast to JAK2-WT, JAK2-V617F clearly lost the favorable interactions between JH1 and JH2 and showed greater conformational changes in the 30 ns MD simulation, as measured by average RMSD (3.8±0.6 Å in WT and 4.4±0.8 Å in V617F) and averaged interaction energy (−279.4±22.1 kcal/mol in WT and −235.4±18.2 kcal/mol in V617F) (Figures S4A–B). In particular, the V617F mutation led to a dramatic conformational rearrangement of the activation loop in JH1, changing from the initially modeled inactive conformation (largely buried) toward a more active conformation (more opened). In JAK2-WT, the activation loop's motions were less pronounced (Figures 6A–B), consistent with previous simulation results based on Kroemer's model [22]. In addition, the fluctuation of the αC helix in JH2 was blocked in JAK2-V617F (Figure S4C), and the V617 mutation rigidifies αC helix in JH2 in the simulation of crystal structure of JH2 [32]. Notably, a favorable salt bridge interaction, involving R588 in the αC helix of JH2 and E1028 in JH1, was broken in JAK2-V617F but remained stable in JAK2-WT (Figure S4D), suggesting that the V617F mutation releases steric constraints with the activation loop in JH1 via trapping the movement of αC helix in JH2.

The V617F mutant did not convert the inactive conformational state of JH1 to an active one during our 30 ns MD simulation (Figures 6). For example, the fluctuation of the αC helix in JH1, a key region for regulating kinase activity, was blocked in both JAK2-V617F and JAK2-WT (Figures 6C–D). In addition, important interfacial contacts between JH1 and JH2, such as V706 and V1033, and L707 and I973, still remained intact in JAK2-V617F (data not shown). Previously, we showed that V617F is only able to hyperactivate full-length JAK2, and a mutant JAK2 lacking the N-terminal FERM domain had similar activities with or without the existence of the V617F mutation [13]. It is likely that our simulation of V617F in the JH1–JH2 complex only represents an early step of hyperactivation, while other JAK2 domains are required for V617F to fully activate JAK2.

### Experimental assessment of the critical binding interface residues

**Figure 7:**
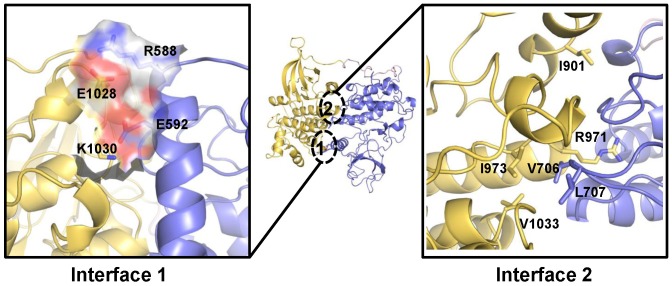
JAK2 JH1-JH2 complex model involves two sets of significant interfacial residues. The JH2 (residues 523 to 816) is shown in blue cartoon while the kinase domain (residues 840 to 1132) is shown in yellow cartoon. The linker loop between two domains is colored pink. Interface 1 shows a site of electrostatic complementarity near the αC helix region of the JH2 and the αEF/αF loop region of kinase domain. The electrostatic complementarity is provided by R588, E592, E1028 and K1030. Interface 2 is composed of mainly hydrophobic residues, especially V706, L707, I901, R971, I973 and V1033.

Our JH1–JH2 model presents unique structural features (Figure 7) that are different from Kroemer's model (Figure S5). Our model is in an anti-symmetric-like and face-to-face domain arrangement, with extensive interactions spanning the αC helix and the αEF/αF loop of both JH2 and JH1. This new dimeric form results in extensive electrostatic (interface 1) and tightly packed hydrophobic interactions (interface 2). Interface 1 is dominated by two inter-domain salt bridge interactions, formed by R588 and E592 in the αC helix of JH2, and E1028 and K1030 in the αEF/αF loop of JH1, respectively. Interface 2 is dominated by strong hydrophobic contacts between V706 and L707 in the activation loop region of JH2 with I973 and V1033 in JH1. Importantly, our model predicts the critical role of residues V706 and L707 in JH2 in stabilizing the inactive conformation of JH1, which could not have been identified in Kroemer's model (Figure S5). In addition, our model indicates that R588 in JH2 forms strong salt bridge interaction with E1028 in JH1 while it interacts with E890 in Kroemer's model, and E592 in JH2 interacts with K1030 in JH1 while it contacts with R893 in Kroemer's model.

To assess our model, we performed computational alanine scanning of the JH1–JH2 interface (Table S1) [33]–[35], and selected corresponding residues for mutagenesis studies. For interface 1, we engineered R588A and E592A in JH2, and the corresponding E1028A and K1030A in JH1 to examine the predicted inter-domain salt bridge interactions. We also made compensatory mutants R588E/E1028R and E592K/K1030E. For interface 2, we engineered V706A and L707A in JH2, and I901A, R971A, I973A and V1033A in JH1 to examine the predicted hydrophobic contacts. We hypothesized that these mutations will directly interrupt favorable interactions between JH2 and JH1, releasing auto-inhibition to activate JH1 kinase activity.

**Figure 8:**
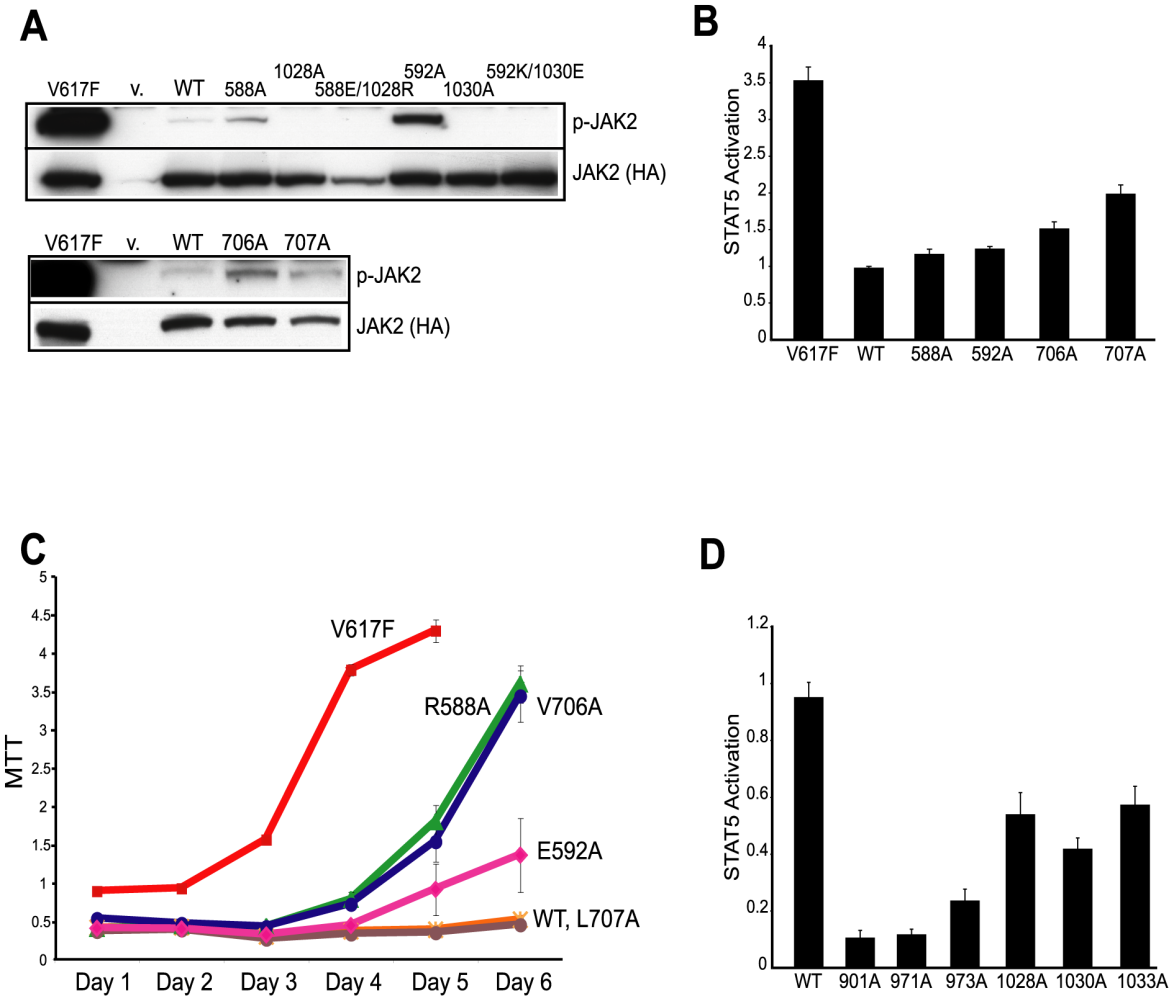
Mutation of interfacial residues changed JAK2 kinase activity. (A) JAK2 mutants showed increased basal kinase activity. Activity of HA-tagged JAK2 mutants was measured by phospho-JAK2 antibodies. Total JAK2 level was measured by anti-HA antibodies. P-JAK2: phosphorylated JAK2. V: vector alone. WT: wild-type JAK2. (B) Hyperactive JAK2 mutants showed increased STAT5 activation. Activation of STAT5 was assessed using flow cytometry with Alexa647-conjugated antibodies to phospho-STAT5. Results were normalized to wild-type JAK2. (C) Hyperactive JAK2 mutants transformed BaF3/EpoR cells into factor-independent growth. Cell growth at each indicated day was measured by MTT assay. JAK2-V617F expressing cells became saturated on Day 5. WT: wild-type JAK2. (D) Mutations in JH1 reduced basal JAK2 kinase activity. Activation of STAT5 was determined as in (B). Results were normalized to wild-type JAK2.

We first examined the effects on JAK2 auto-phosphorylation in HEK293T cells transiently expressing hemaglutinnin (HA)-tagged wild-type or mutated JAK2. As shown in Figure 8A, R588A, E592A, V706A, and L707A were hyperactive compared to JAK2-WT as measured by an antibody specifically recognizing the phosphorylated active form of JAK2. To corroborate these studies, we also examined the ability of these JAK2 mutants to phosphorylate downstream substrate STAT5. Hyperactivation of STAT5 can be observed using antibodies recognizing phosphorylated active form of STAT5 via flow cytometry (Figure 8B). Moreover, we investigated the *in vivo* effect of JAK2 mutants in BaF3/EpoR cells. BaF3/EpoR cells depend on JAK2 activity to proliferate, and expression of JAK2-V617F transforms these cells into factor-independent growth. Consistent with their hyperactivity, R588A, E592A, V706A transformed BaF3/EpoR cells into factor-independent growth, although to a lesser extent than V617F (Figure 8C). L707A was not able to transform BaF3/EpoR cells, despite its hyperactivation. This may be due to the fact that transformation of BaF3/EpoR cells relies on signaling originated from the EpoR-JAK2 complex instead of JAK2 in isolation. L707A may affect the conformation of JAK2 such that although it is hyperactivated, it is less efficient in phosphorylating substrates in the context of a EpoR-JAK2 complex to transform BaF3/EpoR cells. Interestingly, all mutations in the kinase domain (I901A, R971A, I973A, E1028A, K1030A, and V1033A) reduced basal JAK2 kinase activity (Figure 8D). These residues may function in a more complex manner in that they are important both for inhibitory interaction with JH2 and for regulating kinase activity of JH1.

## Discussion

JAK2 plays essential roles in transmitting signals from multiple cytokine receptors, and has emerged as a prominent drug target in hematological malignancies. JAK2 kinase activity is negatively regulated by its JH2 domain, in which a gain-of-function mutation is found in the majority of patients with myeloproliferative neoplasms. Understanding of how the JH2 domain regulates JAK2 kinase activity (JH1) thus is urgently needed. In the absence of full-length JAK2 structures, we developed a model of the JAK2 JH1–JH2 complex using computational modeling. We assessed this model by mutating critical residues in the predicted complex interface in JH2 and showed that they indeed hyperactivated JAK2 kinase activity. Our model requires further experimental validation. Nevertheless, it represents a verifiable working hypothesis that facilitates structure-function interrogation of mechanisms underlying JAK2 signaling. Importantly, our model was built by a novel strategy based on allosteric sites on interacting partners. This step-wise computational strategy we devised may be easily adopted for studying novel protein-protein interactions in a general manner.

Several important lessons were learned from our study. First, our study provides “proof-of-principle” evidence that information on allosteric sites of each interacting partner can be used to guide the generation of protein complex structures. In addition to four models constructed based on available kinase dimeric interfaces, we applied the MutInf algorithm to identify sites exhibited correlated torsional motions in JH1 and JH2 to guide protein-protein docking. The MutInf method applies equilibrium molecular dynamics simulations to identify correlated motions between spatially unrelated residues, so that novel allosteric sites might be identified in an unbiased, statistically robust manner [28]. Detailed analysis of MD simulation trajectories clearly indicates that Model 2, derived from MutInf, was the most energetically favorable and structurally stable model among the six models built. This work represents the first application of using MutInf in a prospective prediction of sites on two protein domains involved in a protein-protein interface.

Second, *a priori* knowledge of the starting conformation for JH1 and JH2 is important for model generation. In contrast to Kroemer's model in which JH2 was built in the inactive conformation, our initial JH1–JH2 model was built such that JH1 is in the inactive conformation and JH2 in the active conformation. This is based on the latest findings that JH2 possesses kinase activity and auto-phosphorylates two JAK2 residues to maintain basal auto-inhibition. In our model, the active conformation of JH2 traps JH1 in an inactive conformation via direct interfacial contacts. Our experimental data that mutating the important interfacial residues V706 and L707 in the JH2 activation loop hyperactivated JAK2 strongly support our model. The importance of V706 and L707 would not have been noticed if JH2 were built in an inactive conformation. It should be noted that although it is hard to argue that JH2 is not in an active conformation as it phosphorylates negative regulatory JAK2 sites in the basal state, the exact conformation of JH2 and JH1 would have to await a crystal structure of full-length JAK2.

Third, the presence of linker loops between JH1 and JH2, and between SH2 and JH2 play a critical role in model construction. We modeled the loops after we determined the best packing mode between JH1 and JH2 – a necessary step in refining the final complex structure. The JH1–JH2 loop reduced the spatial sampling needed in protein-protein docking and restrained the inter-domain arrangement although it remains challenging of loop prediction for those longer than 12 residues [36]. In addition, we also found that the SH2-JH2 linker loop can stabilize the π stacking interaction between residues F617 and F595 in JAK2-V617F. Consistent with our results, mutations in the SH2-JH2 linker loop (the exon 12 mutations), similar to V617F, hyperactivate JAK2 and are found in patients with myeloproliferative neoplasms [12], [31].

Mutating critical interfacial residues in JH2 hyperactivated JAK2 kinase activity, lending strong support to our model. Among these critical residues, R588 was previously identified in our random mutagenesis screen of residues essential for JAK2 auto-inhibition. E592 is adjacent to residue S591, where a S591L mutation was identified in the same random screen [12]. Differ from Kroemer's model, our model predicts that R588 in JH2 forms strong salt bridge interaction with E1028 in JH1 instead of E890 (Figure S5). Importantly, our model predicts the critical role of residues V706 and L707 in JH2 in stabilizing the inactive conformation of JH1, which could not have been identified in Kroemer's model (Figure S5). Surprisingly, mutating the corresponding interfacial residues in JH1, instead of hyperactivating JAK2, resulted in reduced basal JAK2 kinase activity. Among these residues, none are conserved within the kinase family except for I973 (Table S2). These residues thus are not likely to disrupt the kinase fold or directly reduce enzymatic activity. We envision that they serve dual roles in regulating JAK2 kinase activity. First, they interact with JH2 to trap JH1 in an inactive conformation in the basal state. Second, they regulate JH1 activity upon release from JH2. These JH1 residues may control its conformational transition from an inactive to an active state. Alternatively, they may interact with other JAK2 domains such as the FERM domain to activate JH1 activity. Therefore, mutating these residues, although relieves the inhibitory JH1–JH2 interaction, also hinders JH1 kinase activity. Another hypothesis put forth was that dimerization and autophosphorylation of JH2 might be the predominant mechanism to inhibit JH1 [10], [32]. However, how JH2 phosphorylation of negative regulatory sites results in JH1 inhibition remains elusive. Our results and the contribution of the different mechanisms in JH2-mediated JAK2 regulation await confirmation by experimental structures and further experiments.

During the revision of this manuscript, the Hubbard group reported the X-ray structure of the JAK2 JH2 domain [32]. Superimposition of the crystal structure with our modeled complex structure showed that the two structures are well aligned except in the predicted interfacial regions including the αC helix and the activation loop (Figure S6). Both regions are highly flexible in our simulations and in simulation results reported by Hubbard's group. Importantly, our predicted JH1–JH2 interfaces can still be identified, especially for residues E592 and V706, which lends further support to our model. In addition, the JH2 activation loop is less open compared to our modeled structure. Future work will utilize this new crystal structure of JH2 to further refine our JH1–JH2 complex model.

In summary, we hypothesized that the JH1–JH2 interface involves sites on each partner with a high degree of correlated motions with other sites (i.e. potential allosteric sites), and tested this working hypothesis using a hierarchical protein-protein docking and refinement protocol. Our JH1–JH2 model generated from this approach is more energetically favorable compared to those generated in parallel based on available kinase dimeric forms. We then tested our model with prospective mutational analyses. We note that our approach – predicting potential allosteric sites on each partner using MutInf and subsequently adding restraints between these sites to guide protein-protein docking – is particularly novel, and should be useful in predicting interface regions involved in other protein-protein complexes. We expect that our JAK2 JH1–JH2 structure model may facilitate the further exploration of the atomic events of regulatory mechanisms in JAK protein family (structure models available at http://www.huanglab.org.cn/JAK2_MODEL). We also believe that the computational approach we used here will be applicable in predicting novel protein-protein interactions in other systems in general.

## Methods

### Homology modeling of JAK2 kinase (JH1) and pseudokinase (JH2) domains

The JAK2 JH1 domain was crystallized in the active conformation. We modeled the JAK2 kinase domain in the inactive conformation (residues 840–1132) using its active conformation structure as a template (PDB id: 2B7A) [37], with the αC helix (residues 882–928) and activation loop (residues 992–1018) modeled from the inactive conformation of EGFR (PDB id: 2GS7) [15]. The sequence alignment is shown in Figure S7A. The sequences were aligned using ClustalW (version 2.0.5) with protein fast pair-wise alignment using default parameters [38]. The final sequence alignment used in homology modeling was slightly adjusted based on superimposed structures. Homology model was built using the program MODELLER (version 9v7) [39], [40]. The model with top DPOE assessment scores in MODELLER was selected and validated with PROCHECK with an overall G-factor of −0.15 [41]. Ramachandran plot analysis [42] showed that conformations for 87.4% of residues are located in the most favored regions.

Because no structure was available at the time of our study, we generated a homology model for JH2. The sequence of JH2 (residues 545–816) was aligned with all available human kinase domains in the PDB, where its DPG motif was manually aligned with the conserved DFG motif in kinases (Figure S7B). The sequence identity is about 25% on average, while the highest identify is with the kinase domain of PYK2 (28%) and JAK2 (26%). JH1 can interact with JH2, and also phosphorylates the other JH1 in the dimerized receptor complex *in trans*. These results imply conservation of binding characteristics between these two domains. Thus, the crystal structure of the JAK2 kinase domain (PDB id: 2B7A) [37] was chosen as a template to build the homology model of JH2. The Ramachandran plot analysis showed that conformations for more than 87.2% of the residues are located in the most favored regions.

In addition, the linker between JH2 and JH1 (residues 817–839) was modeled by employing the loop modeling in MODELLER. The SH2-Pseudokinase linker region (residues 523–544) was built based on the inactive c-Abl tyrosine kinase structure [43] (Figure S7C). Both linkers were generated after the construction of the JH1–JH2 complex structure.

### Identification of JH1–JH2 interfaces

We employed two independent strategies to identify binding interfaces between JH2 and JH1. One is based on predicted allosteric sites of JH2 and JH1, and the other on available kinase dimeric forms. In the first strategy, we hypothesized that the near-native JH1–JH2 complex interface should connect allosteric sites of the partners identified by a mutual information-based analysis of MD simulations (MutInf) [28]. This method analyzes MD simulation trajectories to calculate the mutual information between pairs of residues's conformations using the distributions of the φ, ψ, and ω torsion angles of the protein backbone and the χ torsion angles of the amino acid side-chains from the MD simulations and then applies tests of significance and statistical corrections to remove noise. We identified candidate allosteric sites for both JH1 and JH2 following the same protocol we previously published [28]. MD simulations were carried out for five parallel systems with different random seeds to improve the conformational sampling. Each copy was minimized in explicit solvent, followed by equilibration at 300 K using constant volume for 10 ps and using a constant pressure [44] of 1 atm for 5 ns. Production simulation was run for 10 ns, with snapshots of the atomic coordinates recorded every 1 ps. A hierarchical clustering protocol using “heatmap” function and a force-directed network diagram [45] in R package (http://www.r-project.org/) was used to cluster the matrix of mutual information between residues to identify groups of residues showing similar patterns of correlations. These highly correlated residues were grouped to identify candidate allosteric sites. Finally, JH1 and JH2 were manually joined at surface regions where their statistically correlated sites could be coupled together to form functional interactions. A similar strategy that predicts allosteric sites using statistical coupling analysis [46] was used to engineer proteins with regulatory activity.

In the second strategy, we constructed JH1–JH2 complex models based on available kinase dimeric forms similar to those in the study performed by Kroemer and coworkers [19], [20]. A total of four diverse JH1–JH2 complex structures were modeled according to the dimer structures of FGFR1 [18], FGFR2 [26], BRAF [29] and RNA-dependent protein kinase PKR [16].

### Hierarchical protein-protein docking procedure

To eliminate the physically unrealistic interactions produced by manual superposition and to explore the localized potential energy surface efficiently, a local protein-protein docking perturbation was performed using RosettaDock program (Rosetta 2.3) [47], [48]. The Rosetta sidechain packing algorithm was applied to allow sidechain flexibility during docking [48]. The docking poses of the JH1–JH2 complex were sampled by spanning a Gaussian random angle of 8° around the axis of the centers and by tilting 8° from the axis after translating JH2 by Gaussian-distributed random distances with a 3 Å standard deviation along the line connecting protein centers and an 8 Å standard deviation in the two perpendicular directions [47]. A total of 10,000 poses were generated for each predetermined dimer configuration.

Subsequently, all the generated docking poses were filtered by the length of the linker between JH2 and JH1 domains (cutoff value of 60 Å). The remaining poses (within 1000) were clustered using NMRCLUST program [49] by computing the root-mean-squared distance (RMSD) of Cα atoms of JH2 after superimposing JH1. Each representative pose from the top 5 clusters was selected for further MD simulation refinement.

The JH1–JH2 complex structure was prepared using Maestro (*Schrödinger* LLC, New York NY). Molecular dynamics was performed by employing the program Desmond 2.2.7.3.0 [50] with OPLS 2005 force field [51] in 0.15 M NaCl [52] and TIP3P explicit water model [53]. The cubic boundary condition was selected and no protein atom was within 10 Å of the edge. The whole system contains about 89,000 atoms and is 98×98×98 Å^3^ in size. The equilibration of solvated system was performed with 2,000 steps of steep descent minimization followed by 3,000 steps of L-BGFS minimization, with 50 kcal·mol^−1^ ·Å^−2^ harmonic position restraints applied to heavy atoms of the solute. The production run was performed in MTK NPT (1 bar, 300 K) ensemble for 10 ns. The cutoffs of short-range electrostatic and Lennard-Jones interactions were 10 Å. Long-range electrostatic interactions were computed by the Particle Mesh Ewald method [54] using 64×64×64 grid with σ = 2.18 Å . The M-SHAKE algorithm [55] was used to constrain all bonds involving hydrogen atoms with the integration step size of 2 fs.

The 10 ns MD simulation was analyzed by root-mean-squared fluctuations of JH2 Cα atoms after superimposing onto the kinase domain (PK_RMSF), buried surface areas (BSA) measured by the program MSMS [56], and hydrophobic contacts and ionic interactions (H/I interactions) in the binding interface calculated by the PIC server [57]. The interaction energy was calculated by subtracting the energies of pseudokinase and kinase domains from the energy of complex (E_bind_ = E_complex_−E_JH1_−E_JH2_) using the Protein Local Optimization Program (PLOP) [58]–[60]. The interaction energy was simplified by accounting the sum of electrostatic (E_ele_) and van der Waals (E_vdw_) interaction terms. Finally, a 30 ns extended MD simulation was performed to further refine the selected near-native JH1–JH2 complex structure after modeling the linkers between JH2 and JH1, and between SH2 and JH2, respectively.

The computational alanine scanning of the JH1–JH2 interface was conducted on the Robetta server [33]–[35] using the refined JH1–JH2 complex structure. The conservation analysis of mutating residues in the kinase domain was carried out on the ConSurf server [61], [62] by collecting 150 JAK2 homologue sequences.

### Immuno-blotting

HA-tagged JAK2 and JAK2 mutants were expressed in the pcDNA3.1 vector. HEK293T cells transiently expressing wild-type or JAK2 mutants were lysed in 1% NP-40 lysis buffer (50 mM Tris/HCl (pH 7.4), 150 mM NaCl and 1% Nonidet P40) with phosphatase and protease inhibitors. The lysates were immunoblotted with antibodies recognizing activated JAK2 (anti-phospho-JAK2, EMD Millipore #07-606, 1∶1000) or HA (Covance #MMS-101P, 1∶1000) [12].

### BaF3/EpoR cell proliferation assay

Wild-type or mutants JAK2 were stably expressed in the MSCV-IRES-CD4 vector in BaF3/EpoR cells. The expression levels of wild-type or mutant JAK2 were similar based on expression of CD4 via flow cytometry (Figure S8). To examine factor-independent growth, cells were washed extensively in RPMI medium with 1% BSA and then grown in RPMI medium with 10% fetal bovine serum without IL-3 as previously described [12]. Cell numbers were determined at days indicated by MTT assay. Cells were seeded in triplicate (10,000/well in 100 µl) in 96-well plates. 15 µl of 3-(4,5-dimethylthiazole-2-yl)-2, 5-diphenyl tetrazolium bromide (MTT; Promega, Madison, WI) was then added to each well to determine live cell numbers according to the manufacturer's instruction.

### Flow cytometry

HEK293T cells were co-transfected with plasmids expressing STAT5 and wild-type or mutant JAK2. 48 hrs post transfection, cells were fixed with 1.6% paraformaldehyde, permeabilized with acetone, washed with staining buffer (PBS with 1% BSA), and stained with Alexa647-conjugated phospho-STAT5 antibodies (BD Biosciences #612599, 1∶50). Fluorescence was determined by flow cytometry on a FACS Calibur (BD Biosciences) and median fluorescence from 10,000 cells was analyzed by FlowJo software [63].

## Supporting Information

Figure S1 **Correlated motions couple distant sites in the active conformation of JAK2 kinase domain identified by MutInf.** (A) The full pairwise matrix of mutual information between residues is shown for the active conformation of the JAK2 kinase domain. (B) A force-directed network diagram is shown for “hub” residues mediating correlations between these sites, highlighting the αC helix (red) and the activation loop and hinge regions (green). (C) The sub-matrix showing only highly coupled residues in the active conformation of JAK2 kinase domain. Strong correlations were observed between the αC helix (red box) with hinge region (green box 1) and activation loop (green box 2), and are shown mapped onto the structure (D) according to Figure S1B.
(TIF)

Figure S2 **The hierarchical clustering and a force-directed network analysis of JAK2 kinase domain in the inactive conformation and the JH2 in the active conformation.** The full pairwise matrix of mutual information between residues is shown the inactive conformation of JAK2 kinase domain (A) and JAK2 JH2 (C). A force-directed network diagram for “hub” residues mediating correlations between sites is shown for the kinase domain (B) and JH2 (D). The two sites highlighted in (B) are the activation loop (green) and the αC helix (red), and in (D) the two sites highlighted are the loop of β7–β8 sheet near the hinge region (green) and the activation loop (red).
(TIF)

Figure S3 **The centroid distance between F595 and F617 in the JAK2-V617F with (red) or without SH2-pseudokinase linker region (blue) in 30 ns of MD shows that the SH2-pseudokinase linker is required to keep these residues in close proximity.**
(TIF)

Figure S4 **The dynamic motions and energetic changes analysis of JAK2-WT and JAK2-V617F in 30 ns MD simulations.** (A) Comparison of the RMSD in 30 ns simulations of JAK2-WT (black) and JAK2-V617F (red), calculated over the Cα atoms of JH2 of JAK2 with respect to the starting structure. (B) Comparison of the interaction energy between the JH2 domain (residues 545–816) and the JH1 domain (residues 840–1132) of JAK2-WT (black) and JAK2-V617F. (C) RMSDs of the αC helix in the JH2 (residues 586–606) show displacement of the helix in JH2-WT but not in V617F mutant. (D) The distance between the Cα atoms of R588 and E1028, representing the distance between the activation loop in JH1 and the αC helix region in JH2, shows more favorable interactions between these two elements in the wild-type kinase than in the mutant.
(TIF)

Figure S5 **Mapping the interfacial residues of our JAK2 JH1-JH2 complex structure onto Kroemer's model.** JH1 (residues 840 to 1123) is shown in yellow cartoon while JH2 (residues 523 to 816) is shown in blue cartoon. The linker loop between two domains is colored in pink. Interface 1 is showing the site of electrostatic complementarity between the two αC helices of the JH1 and JH2, including R588, E592, E890 and R893. The residues of interface 2 in our model (V706, L707) are not located in the protein-protein interfaces of Kroemer's model.
(TIF)

Figure S6 **Detailed view of αC helix and activation loop in the crystal structure (PDB id: 4FVQ) of JAK2 JH2 (Pink) and in our modeled JH2 structure (blue).** The kinase domain is colored in yellow. The originally predicted interfacial residues R588, E592, V706 and L707 are highlighted.
(TIF)

Figure S7 **Sequence alignment used in homology modeling.** (A) The sequence alignment of αC helix and activation loop of JAK2 kinase domain with EGFR (PDB id: 2GS7) was used to build the inactive conformation of the JH1 kinase domain. (B) Sequence alignments are also provided for the JAK2 kinase and JH2 pseudokinase domain and for the SH2 linker (C) (residues 523–544) in JAK2 and ABL (residues 241–260, PDB id: 1OPL).
(TIF)

Figure S8 **JAK2 mutants are expressed at similar levels in Ba/F3 cells.** JAK2 expression was measured by flow cytometry using PE-conjugated anti-CD4 antibodies. Both percentage of cells expressing JAK2 mutants (CD4-positive gate, indicated in each plot) and expression level (CD4 median fluorescence) are similar among Ba/F3 cells expressing each JAK2 mutant.
(TIF)

Table S1 **Hot spot residue prediction on the JAK2 JH1–JH2 interface.**
(DOC)

Table S2 **The conservation analysis of the predicted important interfacial residues in the JH1 kinase domain. The calculated conservation scores of residues on DFG motif are 9.**
(DOC)
