# Supplementary figures and images for: Ab Initio Modeling and Experimental Assessment of Janus Kinase 2 (JAK2) Kinase-Pseudokinase Complex Structure
Source: PLoS Comput Biol. 2013 Apr 4;9(4):e1003022. doi: 10.1371/journal.pcbi.1003022 (PMC3616975; doi:10.1371/journal.pcbi.1003022)

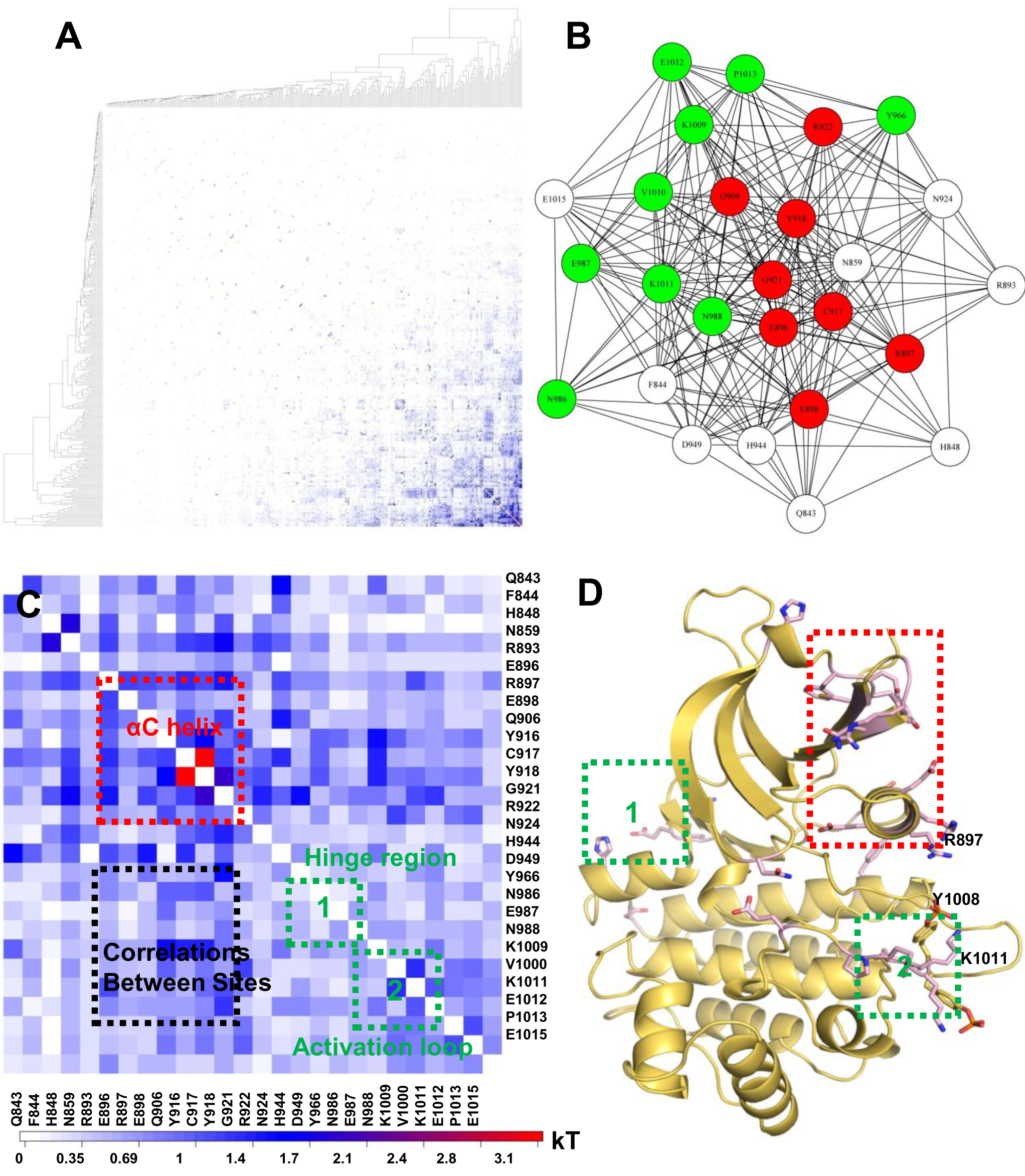

Supplement: Figure S1 — Correlated motions couple distant sites in the active conformation of JAK2 kinase domain identified by MutInf. (A) The full pairwise matrix of mutual information between residues is shown for the active conformation of the JAK2 kinase domain. (B) A force-directed network diagram is shown for “hub” residues mediating correlations between these sites, highlighting the αC helix (red) and the activation loop and hinge regions (green). (C) The sub-matrix showing only highly coupled residues in the active conformation of JAK2 kinase domain. Strong correlations were observed between the αC helix (red box) with hinge region (green box 1) and activation loop (green box 2), and are shown mapped onto the structure (D) according to Figure S1B. (TIF) [file pcbi.1003022.s001.tif]

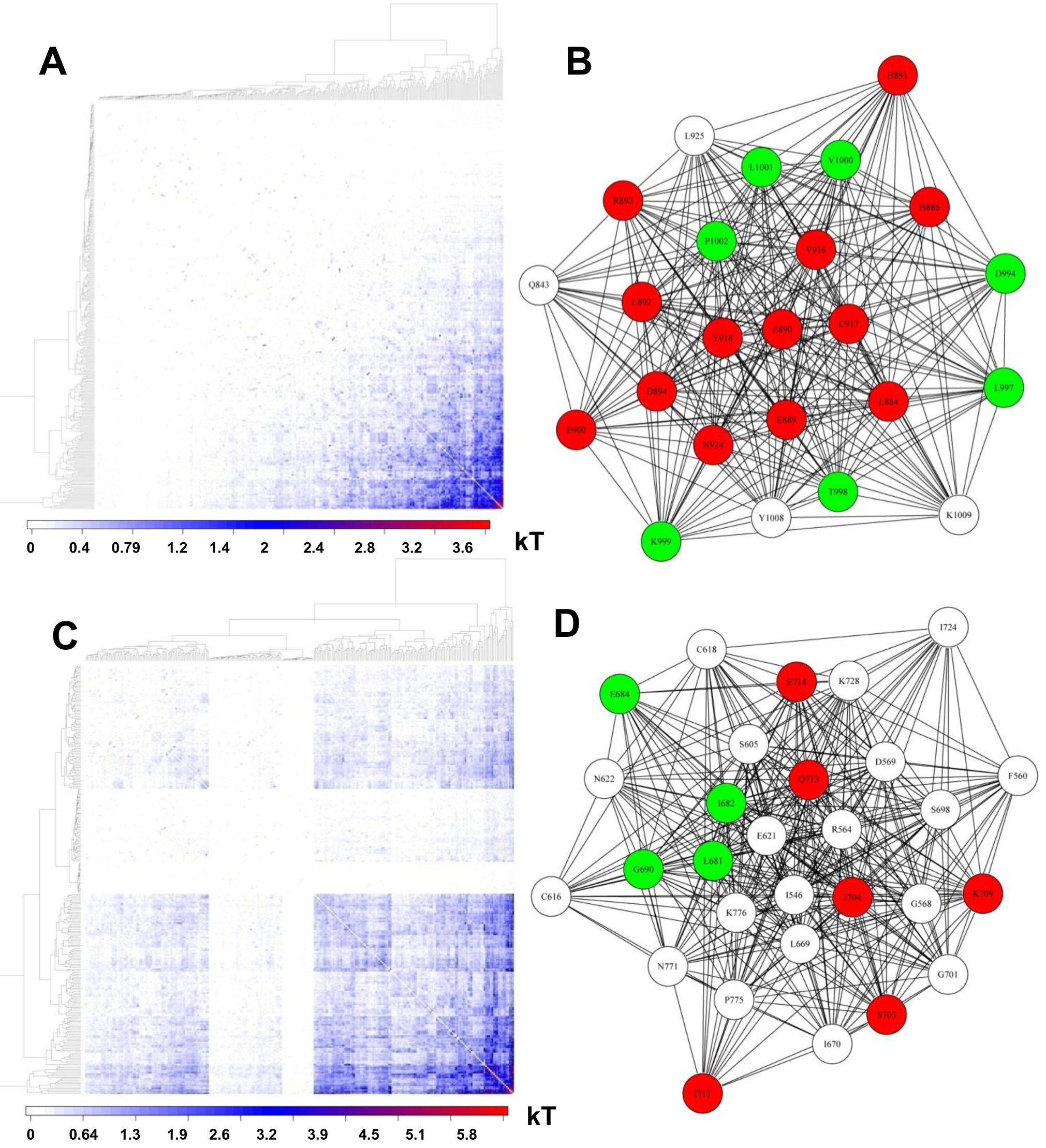

Supplement: Figure S2 — The hierarchical clustering and a force-directed network analysis of JAK2 kinase domain in the inactive conformation and the JH2 in the active conformation. The full pairwise matrix of mutual information between residues is shown the inactive conformation of JAK2 kinase domain (A) and JAK2 JH2 (C). A force-directed network diagram for “hub” residues mediating correlations between sites is shown for the kinase domain (B) and JH2 (D). The two sites highlighted in (B) are the activation loop (green) and the αC helix (red), and in (D) the two sites highlighted are the loop of β7–β8 sheet near the hinge region (green) and the activation loop (red). (TIF) [file pcbi.1003022.s002.tif]

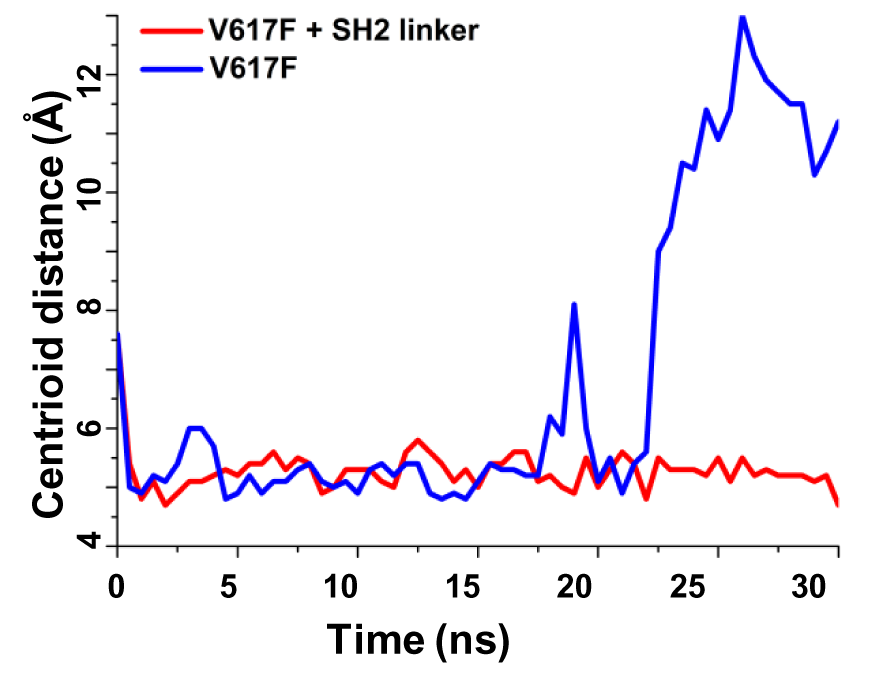

Supplement: Figure S3 — The centroid distance between F595 and F617 in the JAK2-V617F with (red) or without SH2-pseudokinase linker region (blue) in 30 ns of MD shows that the SH2-pseudokinase linker is required to keep these residues in close proximity. (TIF) [file pcbi.1003022.s003.tif]

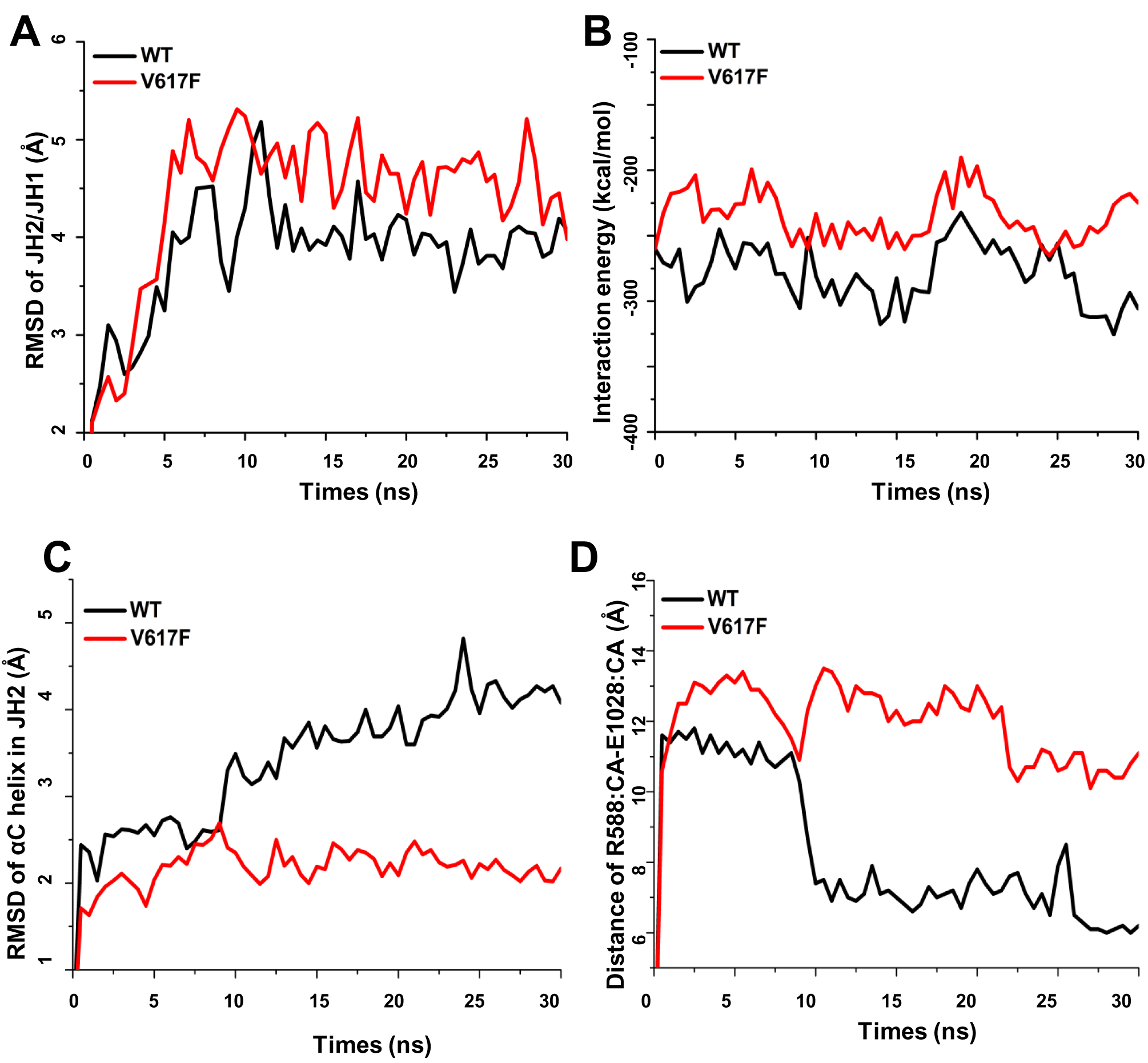

Supplement: Figure S4 — The dynamic motions and energetic changes analysis of JAK2-WT and JAK2-V617F in 30 ns MD simulations. (A) Comparison of the RMSD in 30 ns simulations of JAK2-WT (black) and JAK2-V617F (red), calculated over the Cα atoms of JH2 of JAK2 with respect to the starting structure. (B) Comparison of the interaction energy between the JH2 domain (residues 545–816) and the JH1 domain (residues 840–1132) of JAK2-WT (black) and JAK2-V617F. (C) RMSDs of the αC helix in the JH2 (residues 586–606) show displacement of the helix in JH2-WT but not in V617F mutant. (D) The distance between the Cα atoms of R588 and E1028, representing the distance between the activation loop in JH1 and the αC helix region in JH2, shows more favorable interactions between these two elements in the wild-type kinase than in the mutant. (TIF) [file pcbi.1003022.s004.tif]

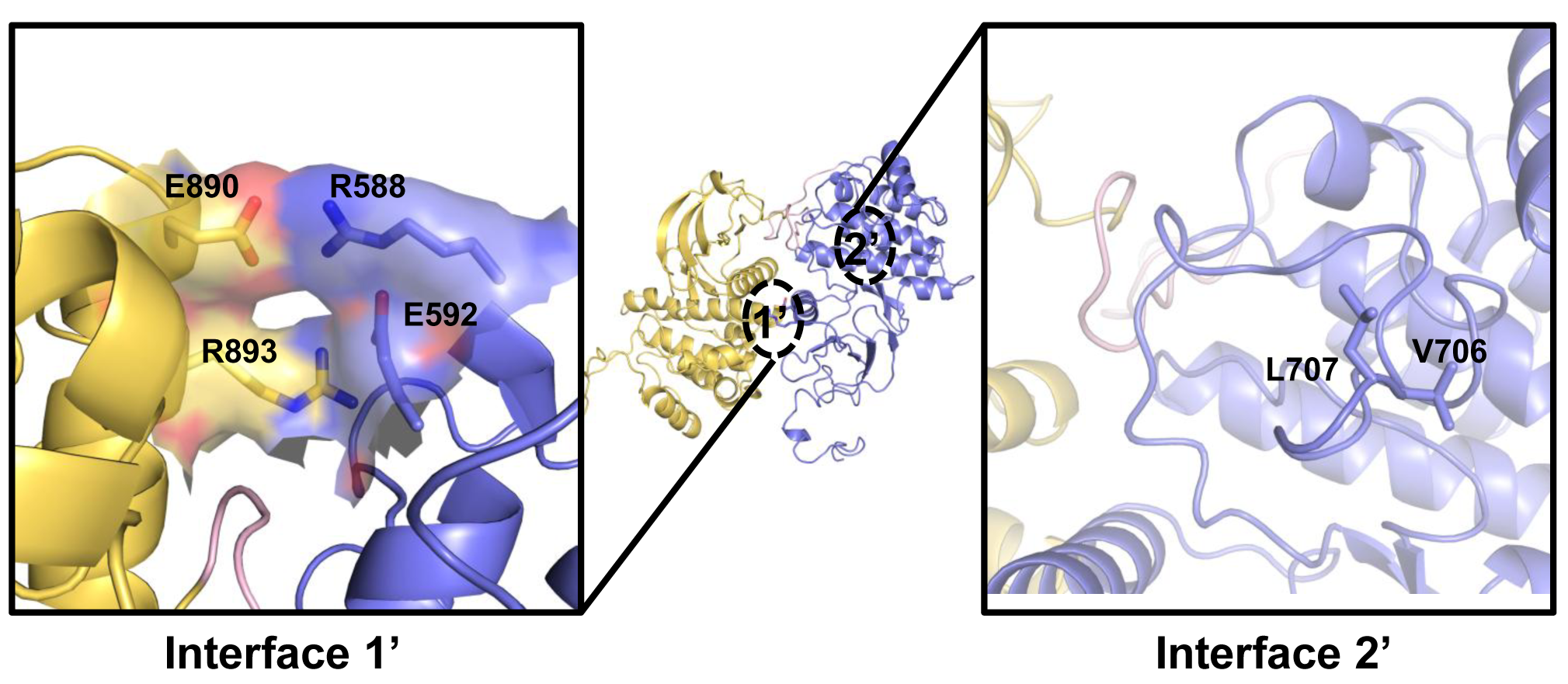

Supplement: Figure S5 — Mapping the interfacial residues of our JAK2 JH1-JH2 complex structure onto Kroemer's model. JH1 (residues 840 to 1123) is shown in yellow cartoon while JH2 (residues 523 to 816) is shown in blue cartoon. The linker loop between two domains is colored in pink. Interface 1 is showing the site of electrostatic complementarity between the two αC helices of the JH1 and JH2, including R588, E592, E890 and R893. The residues of interface 2 in our model (V706, L707) are not located in the protein-protein interfaces of Kroemer's model. (TIF) [file pcbi.1003022.s005.tif]

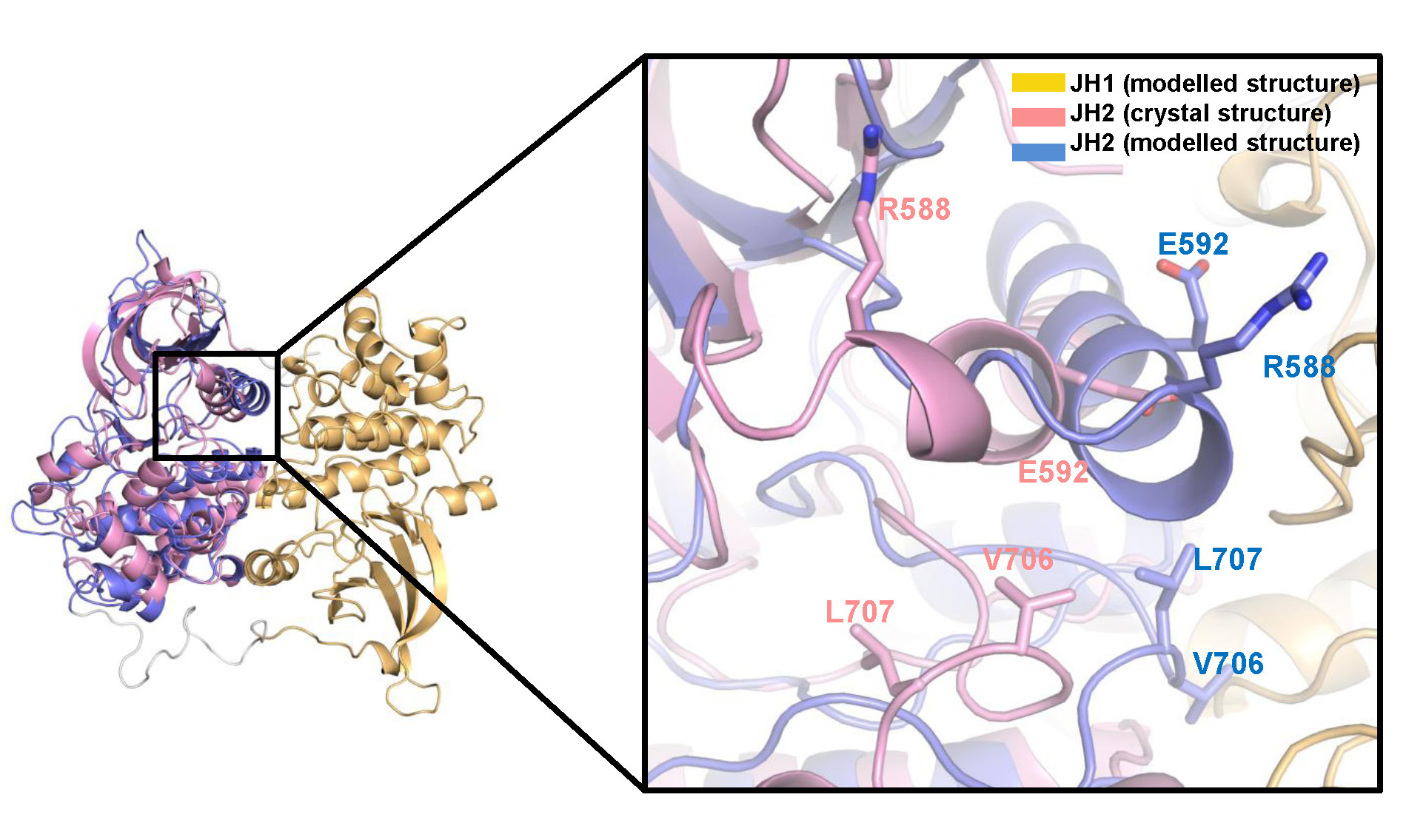

Supplement: Figure S6 — Detailed view of αC helix and activation loop in the crystal structure (PDB id: 4FVQ) of JAK2 JH2 (Pink) and in our modeled JH2 structure (blue). The kinase domain is colored in yellow. The originally predicted interfacial residues R588, E592, V706 and L707 are highlighted. (TIF) [file pcbi.1003022.s006.tif]

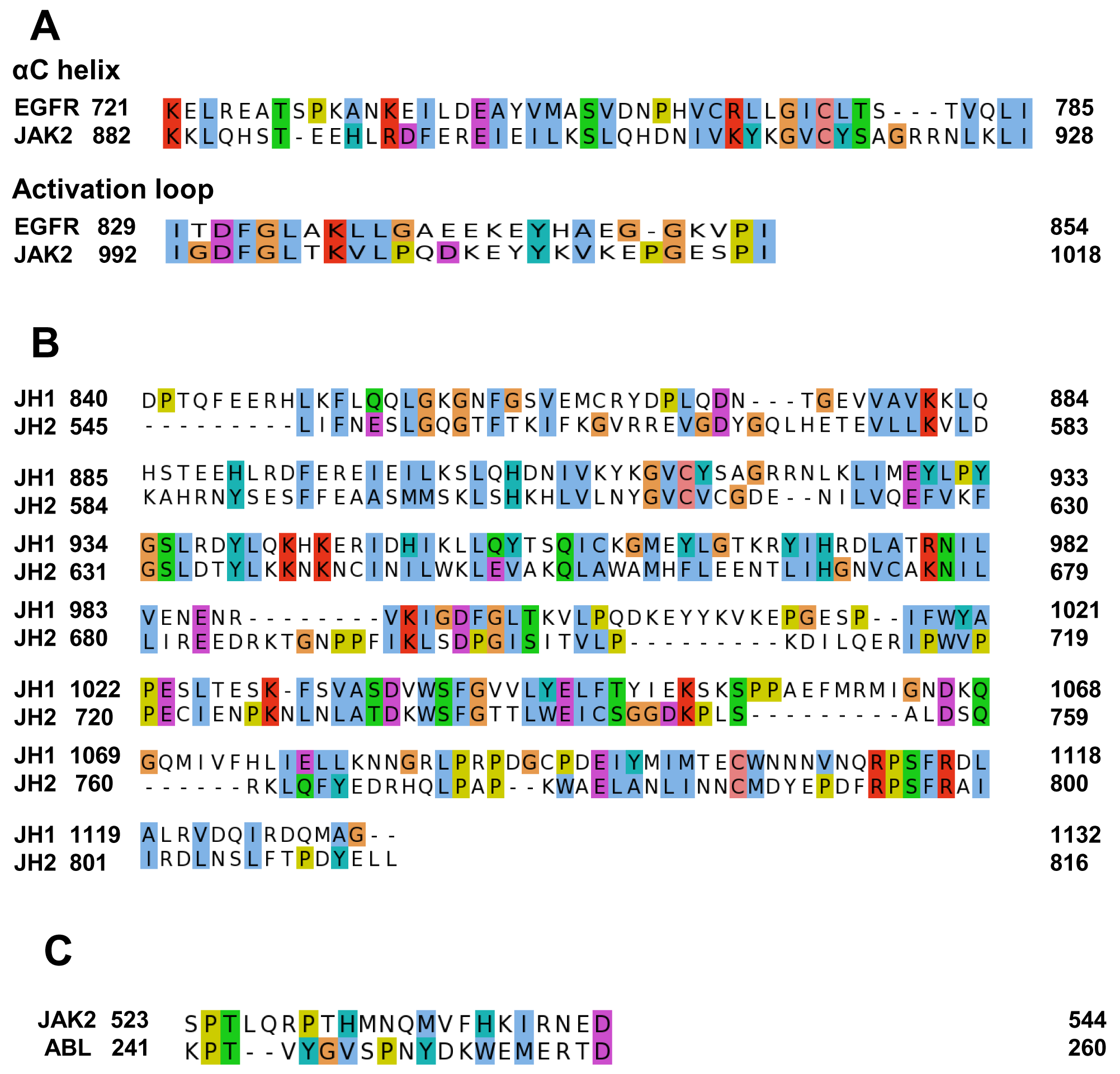

Supplement: Figure S7 — Sequence alignment used in homology modeling. (A) The sequence alignment of αC helix and activation loop of JAK2 kinase domain with EGFR (PDB id: 2GS7) was used to build the inactive conformation of the JH1 kinase domain. (B) Sequence alignments are also provided for the JAK2 kinase and JH2 pseudokinase domain and for the SH2 linker (C) (residues 523–544) in JAK2 and ABL (residues 241–260, PDB id: 1OPL). (TIF) [file pcbi.1003022.s007.tif]

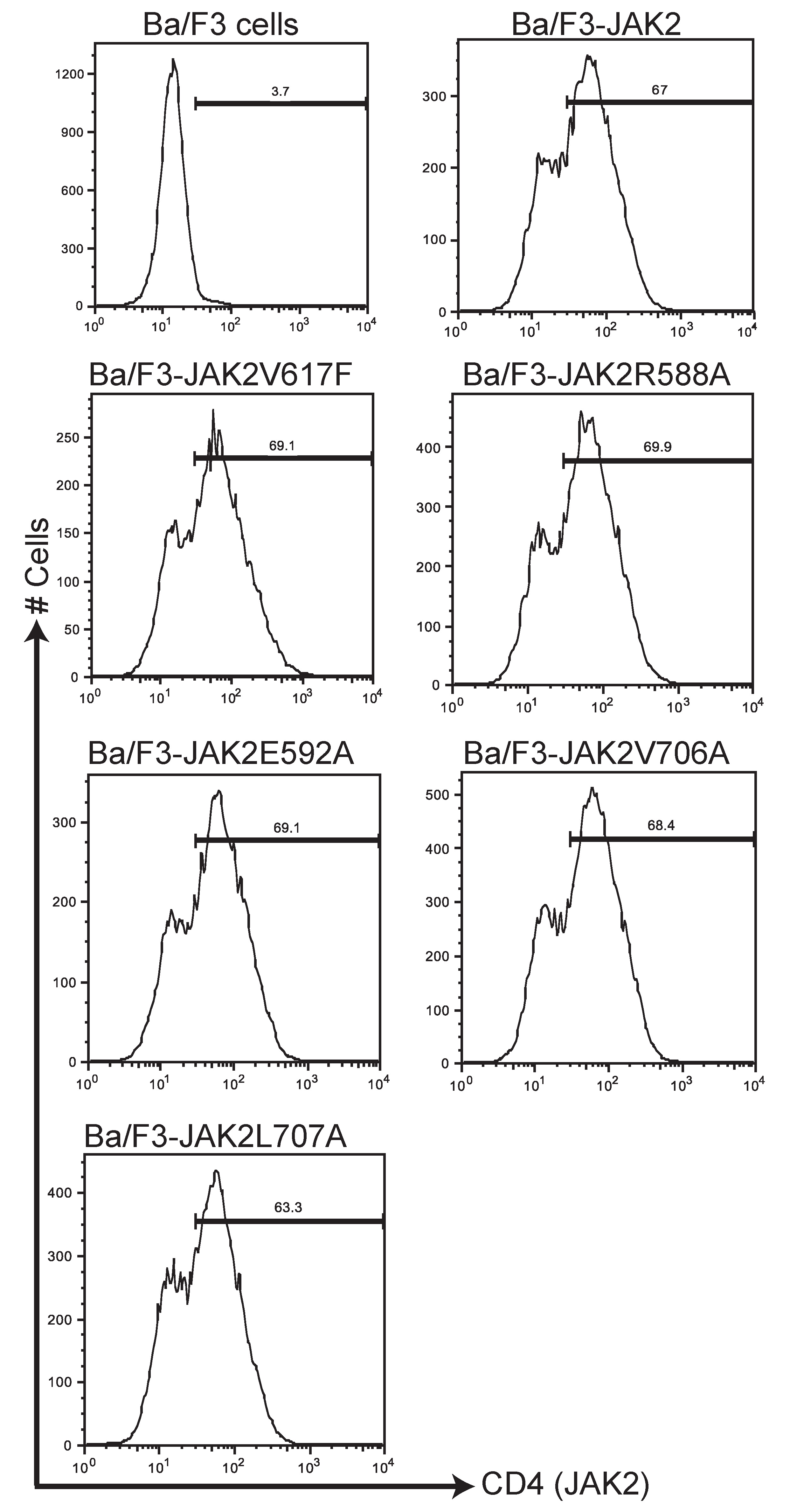

Supplement: Figure S8 — JAK2 mutants are expressed at similar levels in Ba/F3 cells. JAK2 expression was measured by flow cytometry using PE-conjugated anti-CD4 antibodies. Both percentage of cells expressing JAK2 mutants (CD4-positive gate, indicated in each plot) and expression level (CD4 median fluorescence) are similar among Ba/F3 cells expressing each JAK2 mutant. (TIF) [file pcbi.1003022.s008.tif]
