# Supplementary material for: Ab Initio Modeling and Experimental Assessment of Janus Kinase 2 (JAK2) Kinase-Pseudokinase Complex Structure
Source: PLoS Comput Biol. 2013 Apr 4;9(4):e1003022. doi: 10.1371/journal.pcbi.1003022 (PMC3616975; doi:10.1371/journal.pcbi.1003022)
Supplement: Table S1 — Hot spot residue prediction on the JAK2 JH1–JH2 interface. (DOC) [file pcbi.1003022.s009.doc]

| **Residue** | **Chain** | **Calculated ΔΔG_bind_**  **(kcal/mol)** | | **Interface** |
| --- | --- | --- | --- | --- |
| R588 | JH2 | 1.22 | Ⅰ | |
| E592 | JH2 | -0.05 | Ⅰ | |
| E1028 | JH1 | 0.95 | Ⅰ | |
| K1030 | JH1 | 0.62 | Ⅰ | |
| V706 | JH2 | 1.46 | Ⅱ | |
| L707 | JH2 | 0.51 | Ⅱ | |
| I901 | JH1 | 1.3 | Ⅱ | |
| R971 | JH1 | 7.07 | Ⅱ | |
| I973 | JH1 | 0.91 | Ⅱ | |
| V1033 | JH1 | 0.26 | Ⅱ | |
