# Supplementary material for: Ab Initio Modeling and Experimental Assessment of Janus Kinase 2 (JAK2) Kinase-Pseudokinase Complex Structure
Source: PLoS Comput Biol. 2013 Apr 4;9(4):e1003022. doi: 10.1371/journal.pcbi.1003022 (PMC3616975; doi:10.1371/journal.pcbi.1003022)
Supplement: Table S2 — The conservation analysis of the predicted important interfacial residues in the JH1 kinase domain. The calculated conservation scores of residues on DFG motif are 9. (DOC) [file pcbi.1003022.s010.doc]

| **Residue** | **Chain** | **Conservation Score** | **Residue Variety** |
| --- | --- | --- | --- |
| I901 | JH1 | 5 | A,I,L,M,T,V |
| R971 | JH1 | 5 | G,H,K,N,Q,R,S |
| I973 | JH1 | 8 | I,V |
| E1028 | JH1 | 1 | D,E,F,H,L,N,P,Q,R,S,T,Y |
| K1030 | JH1 | 3 | D,E,F,I,K,R,S,T,V |
| V1033 | JH1 | 6 | H,I,L,R,S,T,V,Y |
